# Development of Pelvic Inflammatory Disease after Ectopic Removal

**DOI:** 10.1155/2021/6668299

**Published:** 2021-01-18

**Authors:** Amanda Wang, Sema Hajmurad, Maryam Khan, Sarah Villarreal

**Affiliations:** Department of OBGYN, University of Texas Medical Branch, Galveston, TX, USA

## Abstract

Although ectopic pregnancy and pelvic inflammatory disease (PID) are separately commonly seen in practice, development of PID after surgical removal is rare. Here, we present the case of a 41-year-old female who was admitted for pelvic inflammatory disease diagnosed after laparoscopic salpingectomy for a ruptured ectopic pregnancy. Treatment required drainage of TOAs with interventional radiology and antibiotic treatment. This case report demonstrates how treatment of PID following ectopic pregnancy is complex and may require surgical- or radiology-guided drainage of infection in addition to common antibiotic treatment. Follow-up and duration of treatment are highlighted.

## 1. Introduction

Ectopic pregnancy occurs in 1.5% to 2.0% of all pregnancies [1]. Associated mortality has decreased to 0.5 deaths per 1000 pregnancies [1]; however, despite advancements in treatment, ruptured ectopic pregnancies are the third leading cause of maternal deaths [2]. The fallopian tubes are a common site for extrauterine implantation due to damage by pelvic inflammatory disease (PID) [3, 4], tubal surgery [5], or a prior ectopic pregnancy that significantly increases risk given subsequent scarring and adhesions.

PID is most commonly due to ascending infection from the lower genital tract, with the majority of cases secondary to sexually transmitted infections [6]. However, PID is commonly polymicrobial, including *Gardnerella vaginalis* and obligate anaerobes [7]. Though the presentation is variable, its diagnosis is clinically based on criteria that include lower abdominal pain and at least two of the following: abnormal vaginal discharge, fever, vomiting, menstrual irregularities, urinary symptoms, proctitis symptoms, marked tenderness on examination, palpable adnexal mass, or ESR > 15 mm/hr [8]. If left untreated, PID can cause long-term consequences, including ectopic pregnancy.

PID during pregnancy is extremely rare. It can be difficult to diagnose when ectopic pregnancy is suspected, as many of the symptoms overlap. Unique symptoms may include fever and heterogeneous adnexal masses [9]. One case described a 17-year-old who presented with pelvic pain in the setting of an intrauterine pregnancy who was treated with IV antibiotics with clinical improvement, complicated by incomplete abortion. One larger case study described 57 cases of PID during pregnancy [10], where the most common presenting complaints were cervical motion tenderness and adnexal tenderness, with no significant difference in WBC count or temperature. As heterogenous adnexal masses are seen in both diagnoses, BhCG trends can be used to help differentiate between the two. The recommended antibiotics used to treat PID are all category 2 during pregnancy; however, many are poorly studied during the first trimester [11, 12].

PID after laparoscopic removal of an ectopic pregnancy is similarly rare. A report described a patient who presented with abdominal pain unrelieved by opioids after laparoscopic removal of an ectopic [13]. Under the clinical suspicion of PID, she was empirically treated with clindamycin and gentamicin, which ultimately resolved her symptoms. Another case described PID that developed three months after laparoscopic removal of an ectopic, which resolved with metronidazole and doxycycline [14].

The most common complication of gynecologic surgical procedures is postoperative infection [15]. The unique challenge of gynecologic surgery is introduction of pathogens from the vagina and endocervix. Widespread implementation of antibiotic prophylaxis has significantly reduced infection rates [15]; now, the incidence of surgical site infection (SSI) in the form of pelvic abscesses is less than 1%. Pelvic abscesses most commonly present as the progression of end-stage PID [16].

## 2. Case

A 41-year-old gravida 6 para 2 woman presented with acute onset abdominal pain and dizziness. She denied vaginal bleeding, nausea, vomiting, diarrhea, fever, or chills. The patient's history was significant for a left ectopic pregnancy treated surgically. Last menstrual period was eight weeks prior, and the patient was sexually active without the use of birth control.

She was hypotensive to the 80s/30s mmHG and tachycardic to 100 bpm, and physical exam was notable for a 12-week size uterus and a peritoneal abdomen. Serum hCG was 42,000 mIU/mL. Pelvic ultrasound demonstrated a complex echogenic fluid collection anterior to the uterus with additional complex fluid in the cul de sac. Hypotension and tachycardia worsened, and a dopamine infusion was initiated. Given concern for ruptured ectopic pregnancy, she was taken to the operating room for diagnostic laparoscopy. Laparoscopy revealed a ruptured left tubal ectopic pregnancy with 500 cc of blood in the abdomen. The remaining fallopian tube, uterus, and ovaries appeared normal with no evidence of infection. The patient underwent a left salpingectomy and hemoperitoneum evacuation. Three units of packed red blood cells were given intraoperatively, and she was weaned off pressors before extubation. She had an unremarkable postoperative course and was discharged on postoperative day 1.

Two weeks later, the patient returned with five days of worsening pelvic pain unrelieved by postoperative analgesics, malodorous vaginal discharge, subjective fevers, and nausea/vomiting. Physical exam showed diffuse lower abdominal pain with cervical motion and bilateral adnexal tenderness but no signs of an acute abdomen. The patient was febrile to 101.2°F, tachycardic to 100 bpm, and normotensive. White blood cell (WBC) count was 15.44 × 10∗3/*μ*L, hemoglobin was stable at 10.1 g/dL, and lactic acid was elevated at 10.4 mmol/L. Serum hCG had appropriately dropped to 58mIU/mL. CT abdomen/pelvis showed a distended right fallopian tube with hyperenhancing walls consistent with a pyosalpinx and soft tissue fat stranding in the pelvis consistent with PID. The patient was started on cefoxitin, doxycycline, and metronidazole for presumed PID.

Through hospital day 2, she continued to fever to 103°F. Antibiotic regimen was broadened to vancomycin, cefepime, and metronidazole. Blood and urine cultures showed no growth, and cervical swab was negative for gonorrhea and chlamydia. Interventional radiology (IR) was consulted and on hospital day 4, she underwent a CT-guided drain placement in the right pelvic fluid collection. Her leukocytosis and pain improved, so vancomycin was discontinued. On hospital day 6, the patient continued to fever to 101.2°F, with no output from the drain over 24 hours. Initial drain cultures showed no aerobic or anaerobic growth.

By hospital day 8, the patient was afebrile, but repeat CT abdomen/pelvis showed interval worsening with multiple intra-abdominal/pelvic fluid collections concerning for abscesses, with catheter tip in place. IR then placed a CT-guided drain in the rectovaginal collection; final cultures showed no growth. Antibiotic regimen was tightened to oral doxycycline and metronidazole given improved source control.

In the subsequent day, she remained afebrile with improvement in abdominal pain and was deemed stable for discharge with continued oral antibiotics. The drains were removed two days after. At a follow-up appointment three weeks later, patient was doing clinically well but repeat CT abdomen/pelvis showed an increase in left and right adnexal collection size, so patient was readmitted for further source control.

During her third admission, the patient was afebrile and continued on oral doxycycline and metronidazole. On hospital day 2, she underwent an ultrasound-guided drainage of both the left and right adnexal collections, with near collapse of both collections on postprocedural ultrasound. The clear serous yellow aspirate ultimately showed no growth. Her antibiotics were switched to piperacillin-tazobactam. CT abdomen/pelvis on hospital day 3 showed reappearance of bilateral fluid collections; however, IR determined that the collections were too small for drain placement. Given the patient's lack of symptoms, improving abdominal pain, and afebrile state without leukocytosis, surgical intervention was deemed likely to cause more harm than good. As the patient was subjectively improving, she was discharged on hospital day 3 with oral doxycycline for 10 days. At her last clinic visit, she reported minimal pain, no fevers, and return of regular menstruation.

## 3. Discussion

This discussion summarizes research on available literature on PID, organ space SSI, laparoscopic surgery, laparoscopic surgery complications, ectopic pregnancy, and recurrent pelvic abscess from January 27, 1995, to August 5, 2019. To this date, only two cases have reported PID development after laparoscopic removal of an ectopic pregnancy. Ectopic pregnancy itself is relatively rare, and the incidence of organ space SSI after laparoscopic surgery is <1% [16]. Thus, the incidence of surgical site infection or PID in relation to ectopic pregnancy has been difficult to ascertain.

In this case, diagnosis of PID vs. SSI was difficult, as both are clinical diagnoses that can only be confirmed with biopsy or laparoscopy. She certainly had risk factors for underlying PID given her history of two ectopic pregnancies and pelvic surgery. SSI was also likely given that any residual blood clot in the abdomen would provide an excellent growth media for inoculated bacteria. It is hard to determine whether the patient had underlying infection at the time of surgery, or if the infection was a result of surgical intervention.

Treatment for both infections is similar, consisting of clindamycin or metronidazole with an aminoglycoside, penicillin, or third generation cephalosporin [13]. The antibiotics should then be guided by patient response or culture sensitivities from primary drainage. Our patient's response to treatment also called the diagnosis into question. Her response to treatment was atypically long, considering improvement is generally seen in 1-2 weeks. This patient received appropriate empiric therapy, which was subsequently broadened in addition to primary drainage, as recommended when response is inadequate within 2-3 days [15]. The aspirate from this patient also consistently did not have any microbial growth.

Thus, it is important to consider alternative diagnoses including hydrosalpinx. Given the patient's known history of spontaneous ectopic, it is possible she had an underlying hydrosalpinx in her remaining fallopian tube and may explain why aspirate cultures were unable to grow any organisms. It is also known that hydrosalpinx may be complicated by concurrent or subsequent pelvic infections [17, 18]. Management with appropriate antibiotic coverage is often effective but may require image-guided drainage or surgical interventions [5], as was necessary with this patient.

We must also consider deep septic pelvic thrombophlebitis (SPT). With SPT, patients present with vague symptoms of pelvic pain and fever up to 3 weeks postoperatively [19] but will continue to be febrile despite adequate antibiotic therapy, and blood cultures will be negative. However, SPT is generally treated with a combination of anticoagulation and antibiotic therapy [20], whereas this patient's symptoms resolved after antibiotic treatment and drainage alone.

In summary, this case presents both a challenging diagnosis and subsequent uncommon response to treatment. It emphasizes how alternate diagnoses should always be considered and that treatment must always be individualized. Though PID in the setting of ectopic pregnancy is uncommon, its identification is crucial to appropriate treatment to prevent future complications.

## Data Availability

The data collected for this case contains protected personal health information. This information was retrieved after obtaining written informed consent from the patient discussed in this paper. Thus, we are unable to freely provide access to our data for patient privacy reasons.
